# Development of polymorphic simple sequence repeat markers in *Huperzia serrata* (Lycopodiaceae)

**DOI:** 10.1002/aps3.11273

**Published:** 2019-07-12

**Authors:** Bin Guo, Jing‐yu Ren, Mei‐na He, Kai Yao, Tian‐shu Wang, Li‐qing Wang, Xin Liu, Wei He, Yan‐ping Fu, De‐li Wang, Ya‐hui Wei

**Affiliations:** ^1^ Key Laboratory of Resource Biology and Biotechnology in Western China Department of Life Science Northwest University Xi'an 710069 People's Republic of China; ^2^ Hainan Branch Institute of Medicinal Plant Development Peking Union Medical College and Chinese Academy of Medical Sciences Haikou Hainan 570311 People's Republic of China

**Keywords:** *Huperzia serrata*, Lycopodiaceae, microsatellite primers, transcriptome

## Abstract

**Premise:**

The natural population size of *Huperzia serrata* (Lycopodiaceae) has dramatically decreased and the species has become endangered due to overexploitation. Here, we developed simple sequence repeat (SSR) markers for *H. serrata* to survey both its genetic diversity and population structure.

**Methods and Results:**

Based on 177 individuals, 120 SSR primer pairs were developed and optimized from five regions of the *H. serrata* transcriptomic data. Of these primer pairs, 20 were successfully amplified and 10 showed obvious polymorphism. These polymorphic loci were investigated to study the genetic diversity of *H. serrata*. Two to 11 alleles per locus were identified, the level of observed heterozygosity ranged from 0.00 to 1.00, and the level of expected heterozygosity ranged from 0.19 to 0.79. All loci were successfully amplified in *H. crispata*,* H. sutchueniana*, and *H. selago*.

**Conclusions:**

The 10 polymorphic primer pairs developed here will be valuable for studies of the endangered *H. serrata* and other related species.

*Huperzia serrata* (Thunb.) Trevis., also named qiancengta in China, is a member of the Lycopodiaceae (Almeida, 2016). More than 90% of *H. serrata* species are distributed east of the Hengduan Mountains and south of the Tsinling Mountains–Huai River line, in an area with a subtropical monsoon climate. *Huperzia serrata* is a valuable medicinal plant because it contains the alkaloid huperzine A, which has been shown to effectively attenuate cognitive deficits and has been used for the treatment of Alzheimer's disease (Lei et al., 2015). The rapidly growing demand for natural *H. serrata* and the unrestricted and continuous harvesting has led to the rapid extinction of wild resources. Therefore, many scientists have begun to conduct research on *H. serrata*, including the study of its cultivation and reproduction physiology. However, only a small number of markers (3459 expressed sequence tags) are currently available in the National Center for Biotechnology Information (NCBI), and the current research on *H. serrata* is still limited, especially with regard to molecular information.

Simple sequence repeat (SSR) markers have been widely used as effective genetic markers for plant breeding and genetic applications (Sharma et al., 2009), and have been applied to genetic diversity analysis, genetic map construction, molecular breeding, and germplasm conservation (Kumar et al., 2015). Luo et al. (2010) discovered thousands of SSR loci in *H. serrata* and selected 10 SSR sequences, including several candidate gene‐encoding enzymes involved in bioactive compound biosyntheses, for further detection and verification. However, no optimum SSR loci for the study of genetic diversity and population structure in *H. serrata* have been reported. In this study, microsatellite markers were developed based on the *H. serrata* transcriptome, which will help to investigate the reproductive characteristics of *H. serrata*, evaluate its evolutionary potential, and develop a reasonable strategy for its protection, development, and utilization.

## METHODS AND RESULTS

In this study, 177 *H. serrata* individuals were collected from the Chinese localities of Luan, Enshi, Jizhou, Hanzhong, and Jinping (Appendix S1). Total RNA was extracted from 100 mg of fresh leaves using TRIzol following the instructions of the manufacturer (TIANGEN, Beijing, China). To eliminate potential DNA contamination, we used DNase to purify total RNA following the manufacturer instructions (QIAGEN, Hilden, Germany). RNA purity and concentration were determined by NanoDrop Spectrophotometer (Qubit2.0, Agilent 2100; Shimadzu, Kyoto, Japan). mRNA was isolated using magnetic oligo (dT) beads, and then cut into short fragments using NEBNext Poly(A) mRNA Magnetic Isolation Module according to the manufacturer's instructions (New England Biolabs, Ipswich, Massachusetts, USA). First‐strand cDNA synthesis used random hexamer primers, buffer, dNTPs, and RNase H, and second‐strand cDNA was synthesized by supernumerary DNA polymerase I. The total high‐quality RNA was used to construct the cDNA library. Then, the cDNA library of *H. serrata* was sequenced based on synthesis by sequencing (SBS) technology using the Illumina HiSeq2500 Sequencing platform (Illumina, San Diego, California, USA). After trimming the sequencing linker and primer sequences in reads and after filtering low‐quality data to ensure data quality, high‐quality sequences were de novo assembled into transcript and unigenes. Furthermore, reads were divided into 25‐bp (*k*‐mer) segments using Trinity software (Grabherr et al., 2011). The final assembly was composed of 111,251 unigenes and had an N50 size of 997 bp.

To analyze the genetic diversity of *H. serrata*, annotated unigenes were used to identify SSRs. The identification and localization of SSRs were performed using the MIcroSAtellite Identification Tool (MISA; Thiel et al., 2003). A total of 4395 SSR loci were found by MISA in 3685 unigenes (24.7%), which was higher than previously reported for *H. serrata* (Luo et al., 2010) and for bryophytes such as *Physcomitrella patens* (Hedw.) Bruch & Schimp. (6.3%) (Kobayashi and Morita, 2005). A relatively high frequency of repeats with di‐ and trinucleotides was detected in *H. serrata* (Appendix S1).

Due to short flanking sequences of the SSR loci or inappropriate sequences, only 2064 loci could be used for the design and validation of primer pairs. To investigate the genetic diversity of *H. serrata*, 120 SSR makers were randomly selected and synthesized. DNA amplification was performed with Ex Taq (TaKaRa Biotechnology Co., Beijing, China) following the manufacturer's instructions. PCR amplification conditions were as follows: 95°C for 2 min; 35 cycles at 95°C for 30 s, 45.8–66.8°C (depending on the primer pair) for 30 s, 72°C for 30 s; and a final extension for 5 min at 72°C. PCR products were detected by 1.5% (w/v) agarose gel electrophoresis and 8.0% (w/v) non‐denaturing polyacrylamide gel electrophoresis. SSR primer pairs that produced clear and reproducible polymorphic bands were used to detect alleles via capillary electrophoresis.

There were 20 primer pairs with expected sizes as well as high specificity, amplification efficiency, and repeatability that were successfully amplified by PCR (Table 1). Furthermore, we used the NCBI database to align the selected SSR markers, and annotated their functions separately. Among these 20 primer pairs, 10 primer pairs exhibited monomorphism and were not studied further, and 10 polymorphic primer pairs were used to evaluate the polymorphism information in five populations of *H. serrata*, identifying a total of 72 alleles. The polymorphism information content (PIC) value for SSR primers ranged from 0.313 (primer c52211) to 0.730 (primer c51797) with an average of 0.568; this value was higher than that found in other plants (e.g., *Sesamum indicum* L. [Cho et al., 2011]). The level of expected heterozygosity of the genetic diversity ranged between 0.06 and 0.79 and the level of observed heterozygosity ranged from 0.00 to 1.00 (Table 2). Eight SSR markers had levels of expected heterozygosity above 0.5, indicating a high level of polymorphism in *H. serrata*.

**Table 1:** Characteristics of 20 SSR markers developed for *Huperzia serrata*.

Locus	Primer sequences (5′–3′)	Repeat motif	Allele size range (bp)	*T*_a_ (°C)	*A*	PIC	Putative function [Organism]	GenBank accession no.
c52026.graph_c0	F: TCAAAACCCAACACTCCACA	(AAGG)_6_	138–152	56.8	6	0.476	Light‐harvesting complex [*Selaginella moellendorffii*]	MH298194
	R: TCCTCTCCACACAACCATCA							
c63431.graph_c0	F: TGCTTCATTTCCTCCATCCT	(GCT)_5_	192–195	55.8	2	0.375	Unknown [*Picea sitchensis*]	MH298199
	R: GTGAAGAGGGACAGGCAGAG							
c51797.graph_c0	F: CCTTGTGGGAAAGCGAATAA	(TC)_8_	190–208	55.8	9	0.730	Unknown [*Picea sitchensis*]	MH298193
	R: GCTCGAAACCAACAACGAAT							
c59934.graph_c0	F: CTGCGATCTACAGGCAAACA	(CATC)_5_	256–266	61.8	7	0.601	Predicted protein [*Physcomitrella patens*]	MH298197
	R: AAAACGTTGCCACAAGAAGG							
c52211.graph_c0	F: GCACTCTCTTATTCTGGGCG	(AG)_7_	244–256	56.2	6	0.313	Hypothetical protein SELMODRAFT_17213, partial [*Selaginella moellendorffii*]	MH298195
	R: TGTTTAAGGCCATGAGGAGG							
c65171.graph_c0	F: ATCACGCTCGGAACCACTAC	(CGATCG)_5_	233–257	62.1	11	0.723	Predicted protein [*Physcomitrella patens*]	MH298200
	R: GACCGGGGTCATGATAGAGA							
c50318.graph_c0	F: CCTTTATAGAGTGCAGCGCC	(GT)_8_	252–270	63.8	9	0.618	Phosphoribosylaminoimidazole‐succinocarboxamide synthase, chloroplastic isoform X1 [*Musa acuminata* subsp. *malaccensis*]	MH298192
	R: CATAAGGCAGCACAAGGACA							
c52257.graph_c0	F: GCATGATAAACCAATTCCGTG	(GA)_7_	231–241	52.6	6	0.648	Predicted protein [*Physcomitrella patens*]	MH298196
	R: GACCGGGAAAAGCCATAGAT							
c66382.graph_c0	F: GTTTCTGCTGGATACCTGCC	(GT)_9_	174–196	57.3	10	0.656	Unnamed protein product [*Coffea canephora*]	MH298201
	R: AAATCTGGAGGAGACGACGA							
c60778.graph_c0	F: GGCACATAGAGAAGTAGCGCA	(GA)_7_T(AG)_3_…(CTG)_6_	177–207	53.4	6	0.544	Hypothetical protein AMTR_s00031p00115090 [*Amborella trichopoda*]	MH298198
	R: GGAGTTCTGATTTTCTGCGG							
c38689.graph_c0	F: GGGATCTTGTATAAAGTTCAGTATGC	(GA)_7_	256	58.6	1		Hypothetical protein SELMODRAFT_236822 [*Selaginella moellendorffii*]	MH920530
	R: TCCTGCATGAGCTGTGATTC							
c56357.graph_c0	F: CTTCTCTCGGCAAGCCTTTA	(GT)_7_	195	59.7	1		Hypothetical protein SELMODRAFT_181561 [*Selaginella moellendorffii*]	MH920531
	R: TGACTTAGCGCTTGGGTCTT							
c59441.graph_c0	F: ATGCAGACAGCCTCAATGTG	(CCA)_7_	164	59.8	1		Hypothetical protein SELMODRAFT_136903 [*Selaginella moellendorffii*]	MH920532
	R: CTGCTAGCTTGAAAATGCCC							
c60018.graph_c0	F: GGCAAAAACTGGCAAACAAA	(AG)_8_	226	59.9	1		Predicted protein [*Physcomitrella patens*]	MH920533
	R: ACATACATCACGCACCGAAA							
c61426.graph_c0	F: AGGAAGGGAAGGATTTTGGA	(CCAT)_5_	240	60.2	1		Aminomethyltransferase, mitochondrial [*Beta vulgaris subsp. vulgaris*]	MH920534
	R: CAACCTCCCTTGCTCACCTA							
c62215.graph_c0	F: GTCGTATCGTACCCGTTGCT	(GCC)_6_	246	60.0	1		Hypothetical protein PTT_11286 [*Pyrenophora teres* f. *teres 0‐1*]	MH920535
	R: TCAAGACACACGCCTCACTC							
c62350.graph_c0	F: ACAATCGGACGTTTTGCACT	(GGAA)_6_	214	60.4	1		Hypothetical protein AMTR_s00058p00137050 [*Amborella trichopoda*]	MH920536
	R: ATCGCGTTGCTAGTTCCAAG							
c62412.graph_c0	F: CTGGCAGGTTACACCCTGTT	(AGGC)_5_	177	60.0	1		Hypothetical protein F775_13731 [*Aegilops tauschii*]	MH920537
	R: CTGAGAAAGGGTAAGCGTCG							
c63947.graph_c0	F: CTGCAGCAAACGAAAAATGA	(CGTT)_5_	244		1		Hypothetical protein JCGZ_15140 [*Jatropha curcas*]	MH920538
	R: GTAGAGGCTGATGAGGCCAG							
c64437.graph_c0	F: ACATCCATCTTCCCTTGTGC	(GA)_6_	212		1		Acyl‐protein thioesterase 1‐like [*Prunus mume*]	MH920539
	R: ACGGAATTGAGCTGTGGTTT							
Mean					7.2	0.568		

Note

*A* = number of alleles; PIC = polymorphism information content; *T*_a_ = annealing temperature.

**Table 2:** Genetic characterization of 10 SSRs developed from different populations of *Huperzia serrata*.^a^

Locus	AL (*N* = 19)				HE (*N* = 55)				HJ (*N* = 40)				SH (*N* = 33)				YJ (*N* = 30)			
	*A*	*H*_o_	*H*_e_	HWE	*A*	*H*_o_	*H*_e_	HWE	*A*	*H*_o_	*H*_e_	HWE	*A*	*H*_o_	*H*_e_	HWE	*A*	*H*_o_	*H*_e_	HWE
c50318.graph_c0	6	0.63	0.59	0.000^b^	6	0.84	0.61	0.018	4	0.26	0.43	0.000^b^	4	0.94	0.63	0.000^b^	6	1.00	0.79	0.000^b^
c51797.graph_c0	7	0.68	0.78	0.000^b^	7	0.82	0.64	0.000^b^	6	0.26	0.58	0.000^b^	3	0.97	0.63	0.000^b^	6	1.00	0.79	0.000^b^
c52026.graph_c0	4	0.68	0.57	0.000^b^	4	0.48	0.48	0.010	3	0.23	0.31	0.000^b^	2	0.94	0.51	0.000^b^	3	0.57	0.67	0.000^b^
c52211.graph_c0	2	0.00	0.19	0.000^b^	4	0.02	0.23	0.000^b^	3	0.00	0.42	0.000^b^	2	0.00	0.06	0.000^b^	2	0.00	0.49	0.000^b^
c52257.graph_c0	3	0.16	0.59	0.000^b^	4	0.58	0.71	0.000^b^	5	0.10	0.71	0.000^b^	4	0.00	0.23	0.000^b^	3	0.07	0.52	0.000^b^
c59934.graph_c0	3	0.95	0.62	0.002	5	1.00	0.63	0.000^b^	5	1.00	0.70	0.000^b^	5	1.00	0.71	0.000^b^	3	1.00	0.63	0.000^b^
c60778.graph_c0	4	0.58	0.59	0.220	5	0.77	0.58	0.028	3	0.26	0.23	0.861	3	0.85	0.52	0.001	4	0.87	0.75	0.000^b^
c63431.graph_c0	2	0.49	0.50	0.000^b^	2	1.00	0.50	0.000^b^	2	1.00	0.51	0.000^b^	2	1.00	0.51	0.000^b^	2	1.00	0.51	0.000^b^
c65171.graph_c0	8	0.47	0.76	0.000^b^	5	0.80	0.67	0.000^b^	5	0.28	0.64	0.000^b^	6	0.94	0.67	0.000^b^	4	1.00	0.75	0.000^b^
c66382.graph_c0	4	0.68	0.72	0.000^b^	6	0.82	0.67	0.000^b^	6	0.30	0.54	0.000^b^	4	0.94	0.58	0.000^b^	3	1.00	0.63	0.000^b^

Note

*A =* number of alleles; *H*_e_ = expected heterozygosity; *H*_o_ = observed heterozygosity; HWE = *P* values of exact tests of Hardy–Weinberg equilibrium; *N* = number of individuals.

a Locality and voucher information are provided in Appendix S1.

b Chi‐square test for Hardy–Weinberg equilibrium. Locus showed significant deviations from Hardy–Weinberg equilibrium (*P* < 0.001).

Cross‐species amplification of 10 microsatellite primers was tested on DNA extracts in three related species: *H. crispata* (Ching) Ching, *H. sutchueniana* (Herter) Ching, and *H. selago* (L.) Bernh. Ten loci were successfully amplified in three related species and were shown to be polymorphic (Table 3).

**Table 3:** Cross‐amplification (showing allele size range in base pairs) of the 10 microsatellites developed for *Huperzia serrata* in *H. crispata*,* H. sutchueniana*, and *H. selago*.^a^

Locus	*H. crispata* (*N* = 5)	*H. sutchueniana* (*N* = 5)	*H. selago* (*N* = 5)
c52211.graph_c0	247–254	248–254	254–255
c52026.graph_c0	137–154	138–151	176–184
c63431.graph_c0	193–197	188–197	193–197
c51797.graph_c0	194–207	197–200	193–209
c59934.graph_c0	257–261	257–265	257–270
c65171.graph_c0	234–246	234–235	234–239
c50318.graph_c0	248–278	250–279	248–264
c52257.graph_c0	230–235	231–239	231–241
c66382.graph_c0	180–184	176–184	176–184
c60778.graph_c0	185–188	187–205	187–225

Note

*N* = number of individuals sampled.

a Voucher and locality information are provided in Appendix S1.

## CONCLUSIONS

This study successfully developed 10 polymorphic primers from *H. serrata* and assessed their transferability in related species. The selected polymorphic microsatellites are valuable for the study of wild *H. serrata* resources with regard to its genetic diversity, population structure, and evolution.

## Supporting information


**APPENDIX S1.** Summary of di‐ and trinucleotide repeats in *Huperzia serrata*.

## Data Availability

The raw sequence data reported in this paper have been deposited in the National Center for Biotechnology Information (NCBI) Sequence Read Archive (accession number: SRR8402085). Sequence information of the developed primers has been deposited in NCBI's GenBank, and accession numbers are provided in Table 1.

## Voucher and location information for *Huperzia* species used in this study.

**Table:**

Species	Population code	*N*	Voucher no.^a^^,^ b	Location	Geographical coordinates	Elevation (m)
*Huperzia serrata* (Thunb.) Trevis.	AL	19	NWUHS1001	Luan, Anhui, China	31°28′N, 116°12′E	150
	HE	55	NWUHS1002	Enshi, Hubei, China	30°5′N, 109°11′E	880
	HJ	40	NWUHS1003	Jizhou, Hubei, China	30°08′N, 112°04′E	330
	SH	33	NWUHS1004	Hanzhong, Shaanxi, China	32°30′N, 107°09′E	840
	YJ	30	NWUHS1005	Jinping, Yunnan, China	22°54′N, 103°19′E	1420
*Huperzia crispata* (Ching) Ching	HC	5	NWUHC1001	Jizhou, Hubei, China	30°08′N, 112°04′E	330
*Huperzia selago* (L.) Bernh.	PS	5	NWUHS3001	Longyan, Fujian, China	24°23′N, 115°51′E	460
*Huperzia sutchueniana* (Herter) Ching	HS	5	NWUHS2001	Shizhu, Chongqing, China	27°29′N, 108°39′E	1500

Note

*N* = number of individuals analyzed.

a The samples were stored in the Key Laboratory of Resource Biology and Biotechnology in Western China, Department of Life Science, Northwest University.

b The collector is Jingyu Ren.
