# Supplementary material for: Development of polymorphic simple sequence repeat markers in Huperzia serrata (Lycopodiaceae)
Source: Appl Plant Sci. 2019 Jul 12;7(7):e11273. doi: 10.1002/aps3.11273 (PMC6636615; doi:10.1002/aps3.11273)
Supplement: Supplementary file 1 — APPENDIX S1. Summary of di‐ and trinucleotide repeats in Huperzia serrata. [file APS3-7-e11273-s001.docx]

**APPENDIX S1.** Summary of di- and trinucleotide repeats in *Huperzia serrata.*

| **SSR repeat type** | **No. of unigenes** | **SSRs (%)** |
| --- | --- | --- |
| Dinucleotide |  |  |
| AG/GA/TC/CT | 1546 | 37.5 |
| AC/CA/TG/GT | 205 | 5.0 |
| AT/TA | 78 | 1.9 |
| GC/CG | 27 | 0.7 |
| Trinucleotide |  |  |
| ACG/TGC/CGA/GCT/GCA/CGT | 260 | 6.3 |
| AAG/TTC/AGA/TCT/GAA/CTT | 225 | 5.5 |
| GAC/CAG/CTG/GTC/AGC/TCG | 233 | 5.6 |
| AGG/TCC/CCT/GGA/CTC/GAG | 193 | 4.7 |
| ATG/TAC/CAT/GAT | 82 | 2.0 |
| ACC/TGG/CAC/GTG/CCA/GGT | 60 | 1.5 |
| ACT/TGA/ATC/TAG/TCA/AGT | 53 | 1.3 |
| AAC/TTG/ACA/TGT/CAA/GTT | 43 | 1.0 |
| AAT/TTA/ATA/TAT/TAA | 26 | 0.6 |
| CCG/GGC/CGC/GCG/GCC/CGG | 17 | 0.4 |
